# Randomized, phase II trial to evaluate the efficacy and safety of atezolizumab plus capecitabine adjuvant therapy compared to capecitabine monotherapy for triple receptor-negative breast cancer with residual invasive cancer after neoadjuvant chemotherapy (MIRINAE trial, KCSG-BR18-21)

**DOI:** 10.1186/s12885-025-14673-0

**Published:** 2025-08-09

**Authors:** Jieun Lee, Hee Kyung Ahn, Kyung-Hun Lee, Kyung Hae Jung, Yeon Hee Park, Sung Hoon Sim, Min Hwan Kim, Jee Hyun Kim, Jee Hung Kim, Kyoung Eun Lee, Kyong Hwa Park, Jihong Bae, Moon Hee Lee, Seungtaek Lim, Han Jo Kim, Dae-Won Lee, Jae Ho Jeong, Ji-Yeon Kim, Jin Seok Ahn, Keun Seok Lee, Joohyuk Sohn, Koung Jin Suh, Yoon Jin Cha, Kabsoo Shin, Sung-Bae Kim, Heejung Chae, Gun Min Kim, Seock-Ah Im, In Hae Park, Korean Cancer Study Group (KCSG) Breast Cancer Committee

**Affiliations:** 1 Division of Medical Oncology, Department of Internal Medicine, Seoul St. Mary’s Hospital, College of Medicine, Catholic University of Korea, Seoul, 06591 Korea; 2 Division of Hematology-Oncology, Department of Medicine, Samsung Medical Center, Sungkyunkwan University School of Medicine, Seoul, 06351 Korea; 3 Cancer Research Institute, Seoul National University Hospital, Seoul National University College of Medicine, Seoul, 03080 Korea; 4 Department of Oncology, Asan Medical Center, University of Ulsan College of Medicine, Seoul, 05505 Korea; 5 Center for Breast Cancer, National Cancer Center, Goyang, 10408 Korea; 6 Division of Medical Oncology, Department of Internal Medicine, Yonsei cancer center, Yonsei University College of Medicine, Seoul, 03722 Korea; 7 Department of Internal Medicine, Seoul National University Bundang Hospital, Seoul National University College of Medicine, Seongnam, 13620 Korea; 8 Division of Medical Oncology, Department of Internal Medicine, Gangnam Severance Hospital, Yonsei University College of Medicine, Seoul, 06273 Korea; 9 Department of Hematology and Oncology, Ewha Womans University Hospital, Seoul, 07985 Korea; 10 Division of Hemato-Oncology, Department of Internal Medicine, Korea University College of Medicine, Korea University Anam Hospital, Seoul, 02841 Korea; 11 Division of Medical Oncology, Department of Internal Medicine, Gachon University Gil Medical Center, Incheon, 21565 Korea; 12 Department of Internal Medicine, Inha University Hospital, Incheon, 22332 Korea; 13 Department of Hemato-Oncology, Wonju Severance Christian Hospital, Yonsei University Wonju College of Medicine, Wonju, 26426 Korea; 14 Division of Oncology and Hematology, Department of Internal Medicine, Soonchunhyang University Cheonan Hospital, Cheonan, 31151 Korea; 15 Department of Pathology, Gangnam Severance Hospital, Yonsei University College of Medicine, Seoul, Republic of Korea; 16 Division of Hemato-Oncology, Department of Internal Medicine, Korea University College of Medicine, Korea university Guro Hospital, Seoul, 08308 Korea

**Keywords:** Triple negative breast cancer, Neoadjuvant chemotherapy, Residual disease, Atezolizumab, Capecitabine

## Abstract

Triple-negative breast cancer (TNBC) is an aggressive subtype with poor prognosis, especially in patients with residual disease post-neoadjuvant chemotherapy. This phase II MIRINAE trial (KCSG-BR18-21) evaluates the efficacy and safety of atezolizumab combined with capecitabine versus capecitabine monotherapy as adjuvant treatment in TNBC patients with residual invasive cancer. The primary endpoint is the 5-year invasive disease-free survival (IDFS) rate. Secondary endpoints include IDFS in PD-L1 positive patients, distant relapse-free survival (DRFS), and overall survival (OS). This study addresses the limitations of KEYNOTE-522 by providing data on post-neoadjuvant therapies, potentially establishing a new standard of care for TNBC.

**Trial registration** This trial is registered at ClinicalTrials.gov (NCT03756298).

## Introduction

Triple negative breast cancer (TNBC) is characterized by the absence of estrogen receptor (ER), progesterone receptor (PgR) and human epidermal growth factor receptor 2 (HER2), accounting for 15–20% of all breast cancers [1]. TNBC is more likely to have clinical aggressive features such as early recurrence, drug resistance, and frequent distant metastasis at the diagnosis [1].

In patients diagnosed with early stage TNBC (stage I ~ III), neoadjuvant chemotherapy is often recommended. Achieving a pathologic complete response (pCR) after neoadjuvant chemotherapy is an important prognostic indicator that reflects longer survival and is used as a surrogate endpoint in clinical trials, particularly with TNBC [2]. Conversely, the presence of residual disease after neoadjuvant treatment is strongly associated with poor prognosis and a higher risk of recurrence [3]. Therefore, active administration of neoadjuvant chemotherapy and response-based adjuvant treatment is currently considered as a treatment option in early TNBC [4, 5].

Recently, the integration of pembrolizumab, an immune checkpoint inhibitor (ICI), into neoadjuvant chemotherapy regimens has become a standard approach, significantly improving pCR rates [6]. However, despite these advances, access to ICIs as part of neoadjuvant chemotherapy remains limited for many patients due to barriers such as cost, availability, and regulatory challenges. Moreover, considering the potential risks of irreversible immune-related toxicity of ICI, it is necessary to selectively apply it to patients who would benefit clinically. Consequently, alternative strategies must be explored to improve outcomes for high risk TNBC patients, especially those with residual disease after neoadjuvant chemotherapy. In this context, adjuvant treatment strategies for those patients are critical to addressing this unmet clinical need.

Capecitabine, an oral chemotherapeutic agent, has demonstrated clinical efficacy in the adjuvant treatment of residual TNBC following standard neoadjuvant chemotherapy. The CREATE-X trial, which included patients with HER2 negative breast cancer who did not achieve pCR after neoadjuvant chemotherapy with anthracycline and/or taxane, showed that adjuvant capecitabine improved the 5-yr disease-free survival rate to 74.1% compared to 67.6% in the control group [7]. Notably, in the subgroup analysis, the benefit was more pronounced in the TNBC subtype (HR = 0.58, 95% CI, 0.39–0.87) [7]. Although the KEYNOTE-522 trial demonstrated that neoadjuvant - adjuvant pembrolizumab treatment significantly improved event-free survival (EFS) compared with neoadjuvant chemotherapy alone in patients with early stage TNBC, regardless of the presence of pCR [8]a significant limitation of this study was the use of placebo as the control arm in the adjuvant treatment phase. This approach is not able to answer the critical question of whether pembrolizumab adds any benefit over standard adjuvant treatments such as capecitabine and thus fails to provide comprehensive guidance on optimal post-neoadjuvant adjuvant therapies for patients with residual disease.

Therefore, investigating the efficacy of combining ICIs with chemotherapeutic agents such as capecitabine in the adjuvant setting represents a promising research direction. The rationale for this combination is based on the potential synergistic effects of immunotherapy and chemotherapy to eradicate micrometastatic disease and improve long-term outcomes. Combination of capecitabine with ICI has been evaluated in small phase 2 trials, but only in metastatic setting with no new safety signals [9].

Korean Cancer Study Group (KCSG) breast cancer committee proposed a randomized, phase II trial to evaluate the potential benefit of combining anti-PD-L1 antibody, atezolizumab with capecitabine as adjuvant treatment in TNBC patients with residual disease post-neoadjuvant chemotherapy (MIRINAE study, KCSG BR18-21).

## Objectives and endpoints

### Objectives

The primary objective of the MIRINAE trial is to examine the efficacy of a combination therapy of atezolizumab and capecitabine in patients with TNBC who have either ≥ 1 cm residual invasive breast cancer and/or positive lymph nodes (≥ ypN1) after neoadjuvant chemotherapy. Secondary objectives are to determine the efficacy and safety of the combination treatment in the PD-L1 positive group and all populations. In addition, an exploratory biomarker study will be conducted.

### Endpoints

The primary endpoint is 5-yr invasive disease-free survival (IDFS) rate in the ITT population. Secondary endpoints include the 5-yr IDFS rate in the PD-L1 positive group, distant relapse free survival (DRFS) and overall survival (OS) in all populations and in the PD-L1 positive group. Secondary safety endpoints are the type, grade, frequency and severity of adverse events according NCI-CTCAE version 5.0. Secondary translational endpoints include investigating the relationship between tumor infiltrating lymphocytes (TILs), tumor mutation burden (TMB) and efficacy endpoints (Table 1).

**Table 1:** Objectives and endpoints

Objectives	Corresponding endpoints
Primary objectives	
• To evaluate the efficacy of capecitabine and atezolizumab compared with capecitabine alone in the adjuvant setting (5-yr IDFS)	• 5-yr invasive disease free survival (IDFS) rate in ITT population. IDFS defined as the time from randomization until documented occurrence of ipsilateral or regional invasive breast cancer, distant recurrence, death, contralateral invasive breast cancer, or second primary non-breast invasive cancer
Secondary objectives	
• To evaluate the efficacy of capecitabine and atezolizumab compared with capecitabine alone in the adjuvant setting (5-yr IDFS in PD-L1 positive group, DRFS and OS in all population)	• 5-yr IDFS rate in PD-L1 positive subpopulation • DRFS (distant relapse free survival) defined as the time from randomization until documented distant disease recurrence or death from any cause, whichever occurs first in all patients and in the PD-L1 positive subpopulation • OS (overall survival) defined as the time from randomization to the date of death from any cause in all patients and in the PD-L1 positive subpopulation
• To evaluate the safety and tolerability of atezolizumab and capecitabine compared with capecitabine alone	• Occurrence and severity of adverse events as define d by NCI CTCAE v 5.0
Exploratory biomarker objectives	
• To assess predictive, prognostic, and pharmacodynamic biomarkers associated with efficacy	• Relationship between tumor infiltrating lymphocytes (TILs) and efficacy endpoints • Relationship between tumor mutation burden (TMB) and specific driver gene mutations by FoundationOne assay and efficacy endpoints. • Relationship between immune signature by RNA sequencing and efficacy endpoints.

## Methods/study design

This is a phase II, multi-center, open-label, randomized study designed to evaluate the efficacy and safety of adjuvant treatment with atezolizumab and capecitabine compared with capecitabine alone in patients with TNBC who have either ≥ 1 cm residual invasive breast cancer and/or positive lymph nodes (≥ ypN1) after neoadjuvant chemotherapy.

HER2 and estrogen receptor (ER)/progesterone receptor (PgR) status in patient’s post-neoadjuvant surgical specimens will be used to define TNBC and local laboratory assessment for HER2, ER/PgR will be used. Before randomization, PD-L1 status on tumor infiltrating immune cells (IC) and tumor cells (TC) should be determined by Ventana PD-L1 (SP142) assay. Tumor tissue from very recently obtained (surgical specimens) will be used for PD-L1 testing. In addition, surgically resected tumor tissue will be collected for adjunctive studies. Patients who have consented and are eligible will be randomized in a 1:1 ratio to receive either of the following treatment arms stratified with PD-L1 expression on IC (< 1% vs. ≥ 1%) and yp stage (I vs. II-III).

### Study treatment

**Fig. 1:**
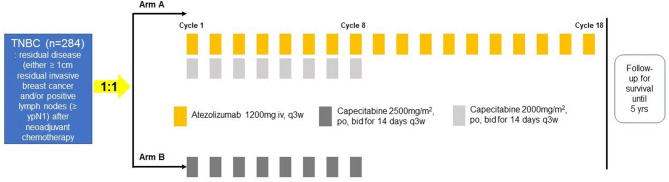
Study scheme

Patients receive atezolizumab plus capecitabine combination after randomization to arm A. The recommended dose of atezolizumab is 1,200 mg administered as an intravenous infusion every 3 weeks for 8 cycles with capecitabine. Then, atezolizumab monotherapy will be continued every 3 weeks up to a total of 18 cycles (for one year of treatment). Capecitabine is administered at a reduced dose (2,000 mg/m2/day, day 1–14, every 3 weeks for 8 cycles) to ensure safety. In the control arm (arm B), patients receive capecitabine monotherapy (2,500 mg/m2/day, day 1–14, every 3 weeks for 8 cycles). After the completion of study treatment, patients will be examined every 3 months for 2 years, every 6 months for 3 years, and then annually follow-up (Fig. 1).

### Study population

This study will enroll patients with TNBC who had residual invasive disease (either ≥ 1 cm residual invasive breast cancer and/or positive lymph nodes (≥ ypN1)) after anthracycline and taxane based neoadjuvant chemotherapy regardless of PD-L1 expression.

#### Key inclusion criteria

1. Male or female ≥ 19 years of age.
2. Patients must have histologically confirmed ER-, PR- and HER2-negative (triple-negative) with residual invasive breast cancer, as defined by the 2010 and 2013 American Society of Clinical Oncology (ASCO) College of American Pathologists (CAP) guidelines, after completion of neoadjuvant chemotherapy; residual disease must be ≥ 1 cm in greatest dimension, and/or have macroscopically positive lymph nodes (ypN+) observed on pathologic exam.
3. Patients must not have metastatic disease
4. Patients must have had neoadjuvant chemotherapy followed by surgery; the recommended neoadjuvant treatment should include 16–24 weeks of a anthracycline and taxane-based third generation chemotherapy regimen as recommended by National Comprehensive Cancer Network (NCCN) guidelines for triple negative breast cancer. Patients who cannot complete all planned treatment cycles for any reason are considered high risk and therefore are eligible for the study if they have residual disease.
5. Patients must have completed their final breast surgery with clear resection margins for invasive cancer and ductal carcinoma in situ (DCIS) within 90 days prior to screening.
6. Patients for whom radiation therapy (RT) to the affected breast or chest wall and regional nodal areas is clinically indicated as per NCCN treatment guidelines, should receive RT before study treatment; RT administered after registration is also allowed.
7. Patients must have resolution of adverse events of the most recent prior chemotherapy and RT to grade 1 or less, except alopecia and ≤ grade 2 neuropathy which are allowed.
8. Patients must not have had prior immunotherapy with anti-PD-L1, anti-PD-1, anti-CTLA4 or similar drugs.
9. Patients must not have had prior capecitabine therapy.
10. Patients must not have had a history of severe allergic, anaphylactic, or other hypersensitivity reactions to chimeric or humanized antibodies or fusion proteins, known hypersensitivity or allergy to biopharmaceuticals produced in Chinese hamster ovary cells or any component of the atezolizumab formulation.
11. Patients must have ECOG performance status < 2.
12. Patients must sign and give written informed consent for this protocol in accordance with institutional guidelines.
13. Patients should have adequate organ function within 21 days prior to the start of study treatment (cycle 1, day1).
14. Women of childbearing potential must have a negative urine or serum pregnancy test within 28 day prior to registration; women/men of reproductive potential must have agreed to use an effective contraceptive method for the course of the study through 150 days after the last dose of study medication.

#### Key exclusion criteria

1. Malignancies other than TNBC within 5 years prior to randomization, with the exception of those with well-differentiated thyroid cancer, carcinoma in situ of the cervix or basal or squamous cell skin cancer.
2. Significant cardiovascular disease, such as New York Hear Association (NYHA) cardiac disease (class II or greater), myocardial infarction within 3 months prior to randomization, unstable arrhythmia, or unstable angina. Patients with a known left ventricular ejection fraction (LVEF) < 50% will be excluded.
3. Patients who have a history of interstitial pneumonitis that required steroids or evidence of active pneumonitis.
4. Known dihydropyrimidine dehydrogenase (DPD) deficiency or history of severe and unexpected reactions to fluoropyrimidine therapy in patients selected to receive capecitabine.
5. Patients who have an active infection requiring systemic therapy.
6. Patients who have active autoimmune disease that has required systemic treatment in past 2 years (i.e., with use of disease modifying agents, corticosteroids or immunosuppressive drugs); patients who are on a stable dose of replacement therapy (e.g., thyroxine, insulin, or physiologic corticosteroid replacement therapy for adrenal or pituitary insufficiency, etc.) are eligible for this study.
7. Treatment with systemic immunostimulatory agents (including but not limited to interferons or IL-2) within 4 weeks or five half-lives of the drug (whichever is shorter) prior to randomization.

### Statistical considerations

#### Estimated number of enrollments

The type I error is controlled for the primary endpoint of 5-yr IDFS rate at alpha = 0.05 (two-sided). The specified sample size will allow for 80% power to detect an improvement in 5-yr IDFS rate from 65% in Arm B to 80% in Arm A. Considering 5% of drop rate, a total of 142 patients will be required in each arm. The test statistic used is the two-sided T-Test. The significance level of the test is 0.05.

#### Efficacy analysis

The ITT population will be used for the primary analysis of 5-yr IDFS rate. An estimate of the 5-yr IDFS rate and its 95% CI will be calculated for each treatment arm. The Chi-test stratified according to tumor PD-L1 status (TC or IC < 1% vs. ≥1%) and pathologic stage (yp stage I vs. II-III) will be used to test 5-yr IDFS rate which are extrapolated from Kaplan-Meier method between treatment groups at a two-sided significance level of 5%. An unstratified chi-test will also be provided.

## Discussion

TNBC is a highly heterogeneous disease, consisting of various subtypes that exhibit differing responses to treatments [10]. In particular, basal-like immune suppressive (BLIS), mesenchymal (MES), and luminal androgen receptor (LAR) subtypes showed lower levels of tumor infiltrating lymphocytes and suppressive immune signatures, which contribute to their reduced responsiveness to immunotherapy [10]. This resistance underscores the critical need for innovative combination strategies to enhance treatment efficacy and improve outcomes in these challenging subtypes [11].

Currently, capecitabine has been validated and accepted as the standard adjuvant treatment for TNBC with residual disease following neoadjuvant chemotherapy [7, 12]. The KEYNOTE-522 trial demonstrated significant benefits in aspect of improving pCR and EFS in stage II-III TNBC. However, there remains an unmet need in non-pCR patients as those with non-pCR in control group received only a placebo instead of the current standard treatment with capecitabine. The study results showed that a 5-year EFS of 62.6% when pembrolizumab was administered to non-pCR patients, suggesting that further improvement is possible if ICIs are combined with other adjuvant agents, such as capecitabine, in this patient population. Nonetheless, the rapidly evolving treatment landscape in early TNBC has made it difficult to conduct new randomized clinical trials that thoroughly evaluate the potential synergistic effects of capecitabine with ICIs, such as anti-PD1/PD-L1 antibodies. Although ongoing phase 3 trials like TROPION-Breast03 include capecitabine plus pembrolizumab as one of the investigator’s choice therapies in the control arm, it is not sufficient to conclude whether there is additional benefit in combining capecitabine and immunotherapy over capecitabine alone [13].

On the other hand, data on the role of ICIs in the adjuvant setting remain insufficient, particularly when ICIs are not included in the neoadjuvant phase. The ALEXANDRA/IMpassion030 phase 3 trial failed to demonstrate the superiority of adding one year of atezolizumab treatment to standard adjuvant chemotherapy after surgery in patients with stage II/III TNBC [14]. Conversely, the A-BRAVE trial, which selectively enrolled higher risk patients who had invasive residual disease after neoadjuvant chemotherapy or who did not receive neoadjuvant chemotherapy but had pathologic stage > IIB, randomized patients to one year of adjuvant avelumab or observation. Although adjuvant avelumab did not significantly improve DFS in high risk TNBC patients, a significant improvement in OS was observed in the avelumab group compared to the observation group with HR of 0.66 [15]. The ongoing SWOG S1418/NRG BR006 trial, a large randomized phase III study, focuses on TNBC patients at higher risk, especially those with residual invasive cancer of primary tumor ≥ 1 cm or positive lymph nodes after neoadjuvant chemotherapy [16]. Although this trial is expected to provide further insight into the role of adjuvant ICI in patients with high risk TNBC, it also has a limitation that the standard adjuvant capecitabine is not incorporated for the control arm. The MIRINAE study address this unmet need by evaluating the combination of atezolizumab with capecitabine in the adjuvant setting. By comparing the clinical outcomes of this combination therapy with capecitabine monotherapy, this study aims to determine whether the addition of atezolizumab in the adjuvant setting can provide significant therapeutic advantage.

Atezolizumab, which targets the PD-L1 pathway, has demonstrated significant clinical activity in both early and metastatic TNBC when used in combination with cytotoxic chemotherapies [17, 18]. The IMpassion-130 trial showed a substantial improvement in progression-free survival (PFS) and OS in patients with PD-L1 positive TNBC when atezolizumab was combined with nab-paclitaxel as a first-line treatment [17]. Additionally, the IMpassion-031 trial further supported the use of atezolizumab, revealing improved pCR rates in the neoadjuvant setting when combined with chemotherapy [18]. Although the accelerated approval of atezolizumab for metastatic TNBC was withdrawn due to the disappointing results of subsequent IMpassion-131[19], it remains valuable to explore the role of atezolizumab in combination with other cytotoxic chemotherapies in different settings.

The MIRINAE trial is a randomized phase II study designed to evaluate whether the addition of atezolizumab to capecitabine in the adjuvant setting improves long-term outcomes in patients with triple-negative breast cancer (TNBC) who have residual disease after standard neoadjuvant chemotherapy. This population represents a group with particularly high risk of recurrence and limited therapeutic options. By incorporating immune checkpoint inhibition into the adjuvant phase and systematically evaluating predictive biomarkers, this study aims to generate clinically meaningful data to inform future treatment strategies for high-risk TNBC.

A notable feature of this study is the decision to perform biomarker analyses using residual tumor tissue obtained after neoadjuvant chemotherapy, rather than pretreatment biopsy specimens. While pretreatment samples may reflect baseline tumor characteristics, they are often unavailable or insufficient in multicenter, real-world settings. More importantly, residual tumors harbor molecular alterations associated with treatment resistance and recurrence, which are highly relevant for guiding adjuvant therapy decisions. Despite concerns about tissue quality following chemotherapy, over 90% of post-neoadjuvant tumor specimens in this trial met the quality control thresholds for next-generation sequencing (NGS), supporting the feasibility of molecular profiling in this context. To ensure consistency and minimize inter-institutional variability, all PD-L1 immunohistochemistry and NGS analyses are conducted centrally in a single reference laboratory.

In conclusion, the MIRINAE trial addresses a critical unmet need in the adjuvant treatment of high-risk TNBC patients with residual disease following neoadjuvant chemotherapy. By evaluating the efficacy of atezolizumab in combination with capecitabine compared to capecitabine alone, this study aims to establish whether the addition of immunotherapy can improve long-term outcomes in this population. The findings are expected to inform future clinical practice and contribute to optimizing adjuvant strategies in TNBC.

## Data Availability

No datasets were generated or analysed during the current study.

## Abbreviations

TNBC: Triple-negative breast cancer
IDFS: Invasive disease-free survival
DRFS: Distant relapse-free survival
OS: Overall survival
ER: Estrogen receptor
PgR: Progesterone receptor
HER2: Human epidermal growth factor receptor 2
pCR: Pathologic complete response
ICI: Immune checkpoint inhibitor
EFS: Event-free survival
KCSG: Korean Cancer Study Group
TILs: Tumor infiltrating lymphocytes
TMB: Tumor mutation burden
IC: Immune cells
TC: Tumor cells
NCCN: National Comprehensive Cancer Network
ASCO: American Society of Clinical Oncology
DCIS: Ductal carcinoma in situ
RT: Radiation therapy
NYHA: New York Hear Association
LVEF: Left ventricular ejection fraction
DPD: Dihydropyrimidine dehydrogenase
BLIS: Basal-like immune suppressive
MES: Mesenchymal
LAR: Luminal androgen receptor
PFS: Progression-free survival
